# Local infrared stimulation modulates spontaneous cortical slow wave dynamics in anesthetized rats

**DOI:** 10.1038/s41598-026-38781-4

**Published:** 2026-02-05

**Authors:** Ágnes Szabó, Richárd Fiáth, Ágoston Csaba Horváth, Péter Barthó, István Ulbert, Zoltán Fekete

**Affiliations:** 1 Faculty of Information Technology and Bionics, Pázmány Péter Catholic University, Práter utca 50/A, Budapest, 1083 Hungary; 2 HUN-REN Research Centre for Natural Sciences, Institute of Cognitive Neuroscience and Psychology, Magyar tudósok körútja 2., Budapest, 1117 Hungary; 3 Department of Neurosurgery and Neurointervention, Faculty of Medicine, Semmelweis University, Amerikai út 57., Budapest, 1145 Hungary

**Keywords:** Infrared neuromodulation, Ketamine/xylazine anesthesia, Local field potential, Multi-unit activity, Slow oscillation, Biological techniques, Neuroscience, Physiology

## Abstract

Cortical slow waves are hallmark oscillations of deep sleep and certain anesthetic conditions, yet the neurobiological mechanisms controlling their dynamics remain incompletely understood. Here, we investigated the effects of local near-infrared (NIR) stimulation on slow-wave activity in ketamine/xylazine-anesthetized rats. Using a silicon-based multimodal optrode, we simultaneously delivered NIR light and recorded local field potentials (LFPs) and multi-unit activity (MUA) across cortical layers in the primary somatosensory (S1Tr) and parietal association (PtA) cortices. NIR stimulation induces local tissue heating, resulting in reproducible and reversible changes in the properties of slow waves. Specifically, up-state durations were shortened, down-states prolonged, and MUA amplitudes during up-states increased, with steeper slopes at state transitions, indicative of enhanced neuronal synchronization. LFP amplitude and spectral changes varied across cortical regions: PtA exhibited increased slow wave (0.5–2 Hz) and high delta (2–4 Hz) band activity, while S1Tr showed a trend toward reduction. Our findings demonstrate that local infrared stimulation can reliably modulate cortical slow-wave dynamics, likely through temperature-mediated changes in neuronal excitability. This approach provides a minimally invasive method for precise, local manipulation of cortical network activity and offers new insights into the biophysical regulation of slow oscillations.

**Supplementary Information:**

The online version contains supplementary material available at 10.1038/s41598-026-38781-4.

## Introduction

Cortical slow waves are the hallmark of natural deep sleep, also known as slow-wave sleep^1–3^. These high-amplitude, rhythmically recurring neural patterns not only characterize deep sleep but also appear under certain anesthetic conditions^2,4–6^. For instance, ketamine/xylazine anesthesia induces a stable and regular slow (~ 1 Hz) rhythm composed of stereotyped slow waves that can be recorded across the entire neocortex^4,7^. In contrast, urethane anesthesia produces neuronal activity patterns more closely resembling those observed during natural sleep^7,8^.

At the cellular level, this slow rhythm manifests as the so-called slow oscillation, characterized by the rhythmic alternation of virtually all cortical neurons between two markedly distinct states: active periods (up-states) followed by silent periods (down-states). During up-states, the membrane potential of neurons is depolarized, resulting in elevated synaptic activity and firing rates, while during down-states, cells display a hyperpolarized membrane potential and completely suppressed spiking activity^2,3^.

Based on our current understanding, the slow oscillation is generated within the thalamocortical network, with the neocortex playing a central role^2,9,10^. In both in vitro and in vivo models, slow waves typically originate in the infragranular layers of the cortex (particularly layer 5) and propagate in both vertical (across layers) and horizontal (across cortical regions) directions^4,10–15^. Although slow waves have a complex propagation dynamic and can initiate from virtually any cortical site, they most frequently start in frontal regions and travel toward posterior areas^16–20^. Additionally, propagation of up-states has also been observed across thalamic nuclei^21^.

Slow-wave activity is closely associated with the consolidation of memories acquired during wakefulness and with the homeostatic regulation of synaptic strengths in cortical networks^22–26^. Furthermore, previous findings suggest that slow waves contribute to the restorative function of sleep by facilitating the clearance of metabolic waste products from the brain accumulated during wakefulness^27,28^.

Previous studies have employed various techniques to perturb the spontaneous slow rhythm in order to better understand its properties, underlying mechanisms, and functional significance. Perturbation methods resulting in rapid and transient changes of cortical activity, such as sensory, electrical, magnetic, or optogenetic stimulation, can reliably alter the ongoing brain state, for instance, by evoking or terminating up-states^11,29–35^.

Other studies assessed the slower and more complex effects of cortical temperature on slow wave dynamics, revealing temperature-dependent changes in both spectral and temporal properties of the slow oscillation^36–38^. These manipulations, however, typically involve warming or cooling relatively large cortical regions, spanning several millimeters.

Given that most slow waves emerging during non-rapid eye movement (NREM) sleep are spatially localized^39,40^, and that local slow waves also occur during rapid eye movement (REM) sleep and wakefulness^40–42^, a more local manipulation of cortical temperature (and with that the indirect modulation of neuronal activity) may uncover additional, previously unrecognized aspects of slow-wave dynamics. One such technique is infrared neuromodulation - a form of photobiomodulation in which pulsed or continuous-wave infrared light is delivered into the brain - producing controlled warming in a spatially confined region of the neural tissue^43^. This photothermal effect can modulate network activity by influencing neural oscillations^44,45^, and by altering the firing rates of neurons, either increasing or decreasing their excitability^46–50^.

In this study, we applied near-infrared (NIR) stimulation to the neocortex of anesthetized rats using a penetrating multimodal probe to investigate how local temperature elevation of the tissue modulates the spectral, temporal and laminar characteristics of slow-wave activity induced by ketamine/xylazine. Cortical electrical activity was recorded simultaneously with NIR stimulation using the same device. The implanted silicon shaft of the probe served as a waveguide, focusing the emitted NIR light in the tissue near the tip of the shaft. Twelve microelectrodes, evenly spaced along the same shaft, were used to record local field potentials (LFPs) and multi-unit activity (MUA) across cortical layers. We analyzed stimulation-induced changes in the duration of up- and down-states, the amplitude of MUA during up-states, the amplitude of slow waves and the amplitude spectrum of low-frequency components (< 4 Hz) of the LFP. These features were compared between supragranular and infragranular layers, as well as between two cortical regions, namely the primary somatosensory cortex and the parietal association cortex.

## Methods

### Silicon-based optical probe

The multimodal probe (optrode) used for near-infrared stimulation and electrophysiological recordings consists of a shaft made from p-type single-crystalline silicon^49–51^. The shaft has a length of 5 mm and a cross-sectional area of 170 μm × 200 μm, and functions both as a waveguide and a linear electrode array. To reduce tissue damage during insertion, the shaft is tapered with a tip angle of 2α = 30°. NIR light is emitted from this sharp tip. The shaft includes 12 square-shaped recording sites (30 μm × 30 μm) arranged linearly with an inter-site spacing of 100 μm, covering a vertical area of approximately 1.1 mm. The first site is positioned 200 μm from the tip. The electrode wiring is composed of platinum (Pt), and the recording sites are coated with an additional porous Pt layer to reduce their electrical impedance to 89.6 ± 43.1 kΩ measured at 1 kHz^50^.

### Animal surgery and optrode implantation

All experiments were conducted in accordance with the EC Council Directive of September 22, 2010 (2010/63/EU), and all procedures were reviewed and approved by the Animal Care Committee of the HUN-REN Research Centre for Natural Sciences and the National Food Chain Safety Office of Hungary (license number: PE/EA/672-6/2021). The study was carried out in compliance with the ARRIVE guidelines. All experiments were conducted during the resting period (light phase) of animals, when time spent in slow-wave sleep, and consequently the density of naturally occurring slow waves, is highest^52^.

For acute in vivo experiments, adult Wistar rats were used (*n* = 8; weight: 331.25 ± 84.35 g, mean ± standard deviation; gender balanced). Surgical procedures, probe implantation, and electrophysiological recordings were performed similarly to our previous studies^4,46,53,54^. Briefly, anesthesia was induced via intraperitoneal injection of ketamine (75 mg/kg) and xylazine (10 mg/kg). To maintain a stable and regular cortical slow oscillation throughout the experiment, supplementary doses of ketamine/xylazine were administered intramuscularly at hourly intervals. A homeothermic heating pad connected to a temperature controller (Supertech, Pécs, Hungary) was used to maintain physiological body temperature. The head of the anesthetized rat was secured in a stereotaxic frame (David Kopf Instruments, Tujunga, CA, USA), after which the skin and the connective tissue were removed from the top of the skull.

In four animals, a cranial window measuring approximately 10 mm × 5 mm was drilled over the left brain hemisphere, centered around the parietal association cortex (PtA; anterior-posterior [AP]: +1 mm to -9 mm; medial-lateral [ML]: 0.5 mm to 5.5 mm; coordinates relative to bregma^55^. This large craniotomy was required to accommodate a flexible micro-electrocorticography (µECoG) array placed on the exposed cortical surface^54,56^. This device was used to collect electrophysiological data for a separate study. The 32-channel µECoG array featured a circular opening (800 μm diameter) through which the optrode was implanted for infrared stimulation and intracortical recording. In the remaining four animals, a smaller craniotomy with a size of 3 mm × 3 mm was prepared (AP: -1.5 mm to -4.5 mm; ML: 1.5 mm to 4.5 mm, with respect to bregma), targeting the trunk region of the primary somatosensory cortex (S1Tr) for optrode insertion. The data obtained from these rats are part of a larger dataset collected in one of our previous studies^46^.

**Fig. 1:**
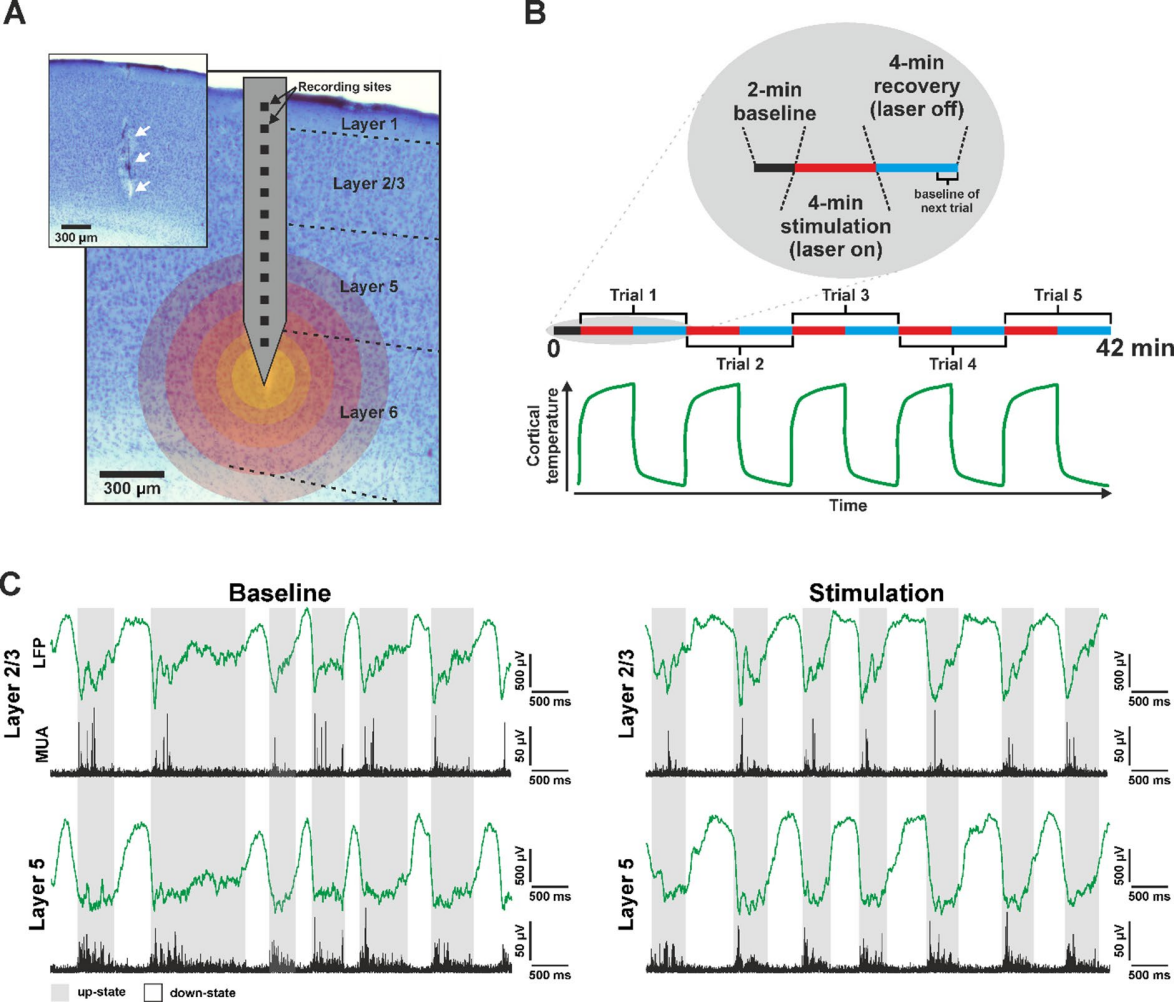
(**A**) Experimental setup. Schematic illustration of the silicon-based optical probe (optrode) overlaid on a Nissl-stained coronal brain section containing the optrode track (indicated by white arrows in the inset). Boundaries of cortical layers are marked with dashed black lines. Colored circles overlaid on the image indicate part of the cortical area affected by infrared stimulation. Lighter colors closer to the optrode tip indicate a higher increase in cortical temperature during stimulation. (**B**) Schematic illustration of the infrared stimulation protocol. In 2 out of the 8 rats, stimulation was applied only for 2 min. Bottom: schematic plot showing the time course of cortical temperature during the stimulation protocol. (**C**) Representative five-second local field potential (green) and multi-unit activity (black) traces recorded from layer 2/3 (top) and layer 5 (bottom) of the neocortex during the baseline period (last minute of an OFF period, left) and during infrared stimulation (last minute of an ON period, right) from a single rat. Gray shading indicates up-states, while white regions correspond to down-states.

At the insertion site, the dura mater was carefully removed using a 34-gauge needle to minimize cortical dimpling during optrode implantation. The optrode was mounted on a motorized stereotaxic micromanipulator (Robot Stereotaxic, Neurostar, Tübingen, Germany) and inserted perpendicularly to cortical layers to a depth of 1.2 mm with a slow rate (2–20 μm/s) to reduce the insertion-related mechanical damage to the tissue^57^. After reaching the target depth, the tip of the optrode was located in infragranular cortical layers (bottom of layer 5, or layer 6; Fig. 1A). The tip position of optrode was chosen based on previous findings indicating a key role for deep layers in the initiation and maintenance of up-states^4,10–15^. To prevent dehydration of the cortical tissue, room temperature physiological saline solution was regularly applied to the brain surface. Since topical application of room temperature saline can transiently reduce tissue temperature and potentially interact with the heating effects of local infrared stimulation, no saline was applied during the infrared stimulation protocol. Furthermore, following saline application, cortical temperature rapidly returns to baseline (within ~ 3 min) and is therefore expected to have a negligible impact on the results^36^. At the end of the experiments, rats were administered an overdose of ketamine/xylazine. Subsequently, animals were either transcardially perfused (*n* = 5) or euthanized by an overdose of isoflurane, with death confirmed by cessation of respiration and heartbeat (*n* = 3).

### In vivo electrophysiological recordings

Cortical electrical activity was collected with the optrode using an Intan RHD2000 electrophysiological recording system (Intan Technologies, Los Angeles, CA, USA) equipped with a 32-channel headstage. Wideband (0.1–7500 Hz) continuous signals were acquired at a sampling rate of 20 kHz per channel (30 kHz per channel for one animal) with 16-bit resolution. For the four animals with the µECoG array, a second 32-channel headstage was used to record ECoG signals; however, this data was not analyzed in the present study. A stainless-steel needle inserted in the nuchal muscle of the animal served as both the reference and ground electrode during recordings.

### Infrared neuromodulation

NIR light at a wavelength of 1550 nm was delivered using a fiber-coupled laser diode with a maximum output power of 70 mW (LPSC-1550-FG105LCA-SMA, Thorlabs GmbH, Bergkirchen, Germany). The laser was driven at a constant current of 400 mA DC, supplied by a Keithley 2611B SYSTEM SourceMeter (Keithley Instruments Inc, Cleveland, OH, USA). Two slightly different stimulation protocols were applied during the experiments. Following a 2-minute-long baseline recording period, the laser diode was activated for either 2 min (*n* = 2 rats) or 4 min (*n* = 6 rats), referred to as ON period. NIR stimulation results in an estimated temperature increase of approximately 4.5 °C and 4.8 °C for 2-min and 4-min ON periods, respectively (based on in vitro measurements reported in our previous study^46^; the recordings from S1Tr analyzed here were collected during that study). Each ON period was followed by a 4-minute OFF period, during which the laser diode was turned off, allowing the tissue and the temperature to recover (Fig. 1B). This ON-OFF cycle was repeated five times consecutively, resulting in recordings with a duration of either 32 min (*n* = 2 rats) or 42 min (*n* = 6 rats). Repetitions will be referred to as trials. The onset of ON and OFF periods was synchronized with the electrophysiological recordings using trigger signals generated by the same Keithley instrument.

### Histology

To visualize the track of the optrode in the brain tissue and to identify cortical layers (Fig. 1A), we followed a histological procedure described previously^4,57^. In short, a subset of rats (*n* = 5) was deeply anesthetized with ketamine/xylazine after neural data collection and transcardially perfused with 100 ml physiological saline solution followed by 250 ml of a fixative solution containing 4% paraformaldehyde in 0.1 m phosphate buffer (pH 7.4). The fixed brains were stored at 4 °C overnight. Subsequently, the brain tissue was cut into 60-µm-thick coronal sections using a vibratome (Leica VT1200, Leica Microsystems, Wetzlar, Germany). After washing in 0.1 M phosphate buffer, the sections were transferred to a Petri dish containing gelatin, mounted onto microscopic slides, and air-dried. The slides were then processed for cresyl violet (Nissl) staining, dehydrated in xylene, and cover-slipped using DePex (SERVA Electrophoresis, Heidelberg, Germany). Finally, Nissl-stained sections containing the optrode tracks were photographed under a light microscope (Leica DM2500, Leica Microsystems) equipped with a digital camera (DP73, Olympus, Tokyo, Japan). The track of the optrode was clearly visible in the brain sections due to the relatively large cross-sectional area of its silicon shaft.

### Data analysis

**Fig. 2:**
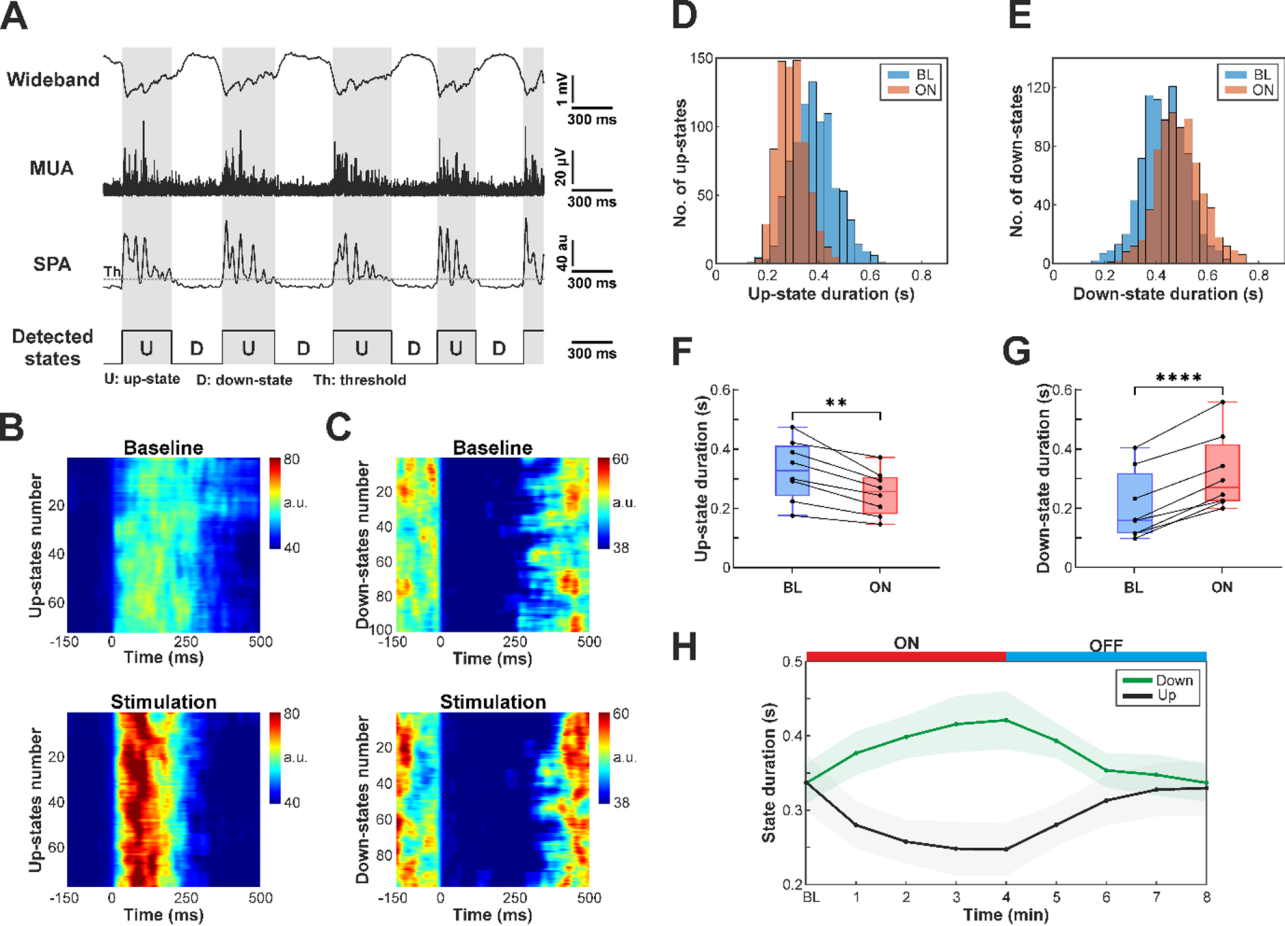
(**A**) Schematic illustration demonstrating the method used for detection of up- and down-state onsets. MUA, multi-unit activity; SPA, smoothed population activity. (**B**,**C**) Color maps of short segments of SPA traces aligned to the detected up-state (**B**) and down-state (**C**) onsets (time point zero), ordered by occurrence. The color maps display SPA traces extracted during the last minute of the OFF period (baseline, top) and the last minute of the ON period (stimulation, bottom) of a single trial. (**D**,**E**) Representative distributions of up-state (**D**) and down-state (**E**) durations during baseline (last two minutes of OFF periods; blue) and stimulation (last two minutes of ON periods; red) periods. (**F**,**G**) Distribution of mean up-state (**F**) and down-state (**G**) durations across all rats during baseline (BL) and the last minute of stimulation (ON) period. Each dot represents an individual rat; lines connect repeated measures. **, *p* < 0.01; ****, *p* < 0.0001. (**H**) Changes in mean up-state (black) and down-state (green) durations during stimulation trials. The red and blue horizontal bars indicate the stimulation (ON) and recovery (OFF) periods, respectively. The shaded areas indicate standard error.

**Fig. 3:**
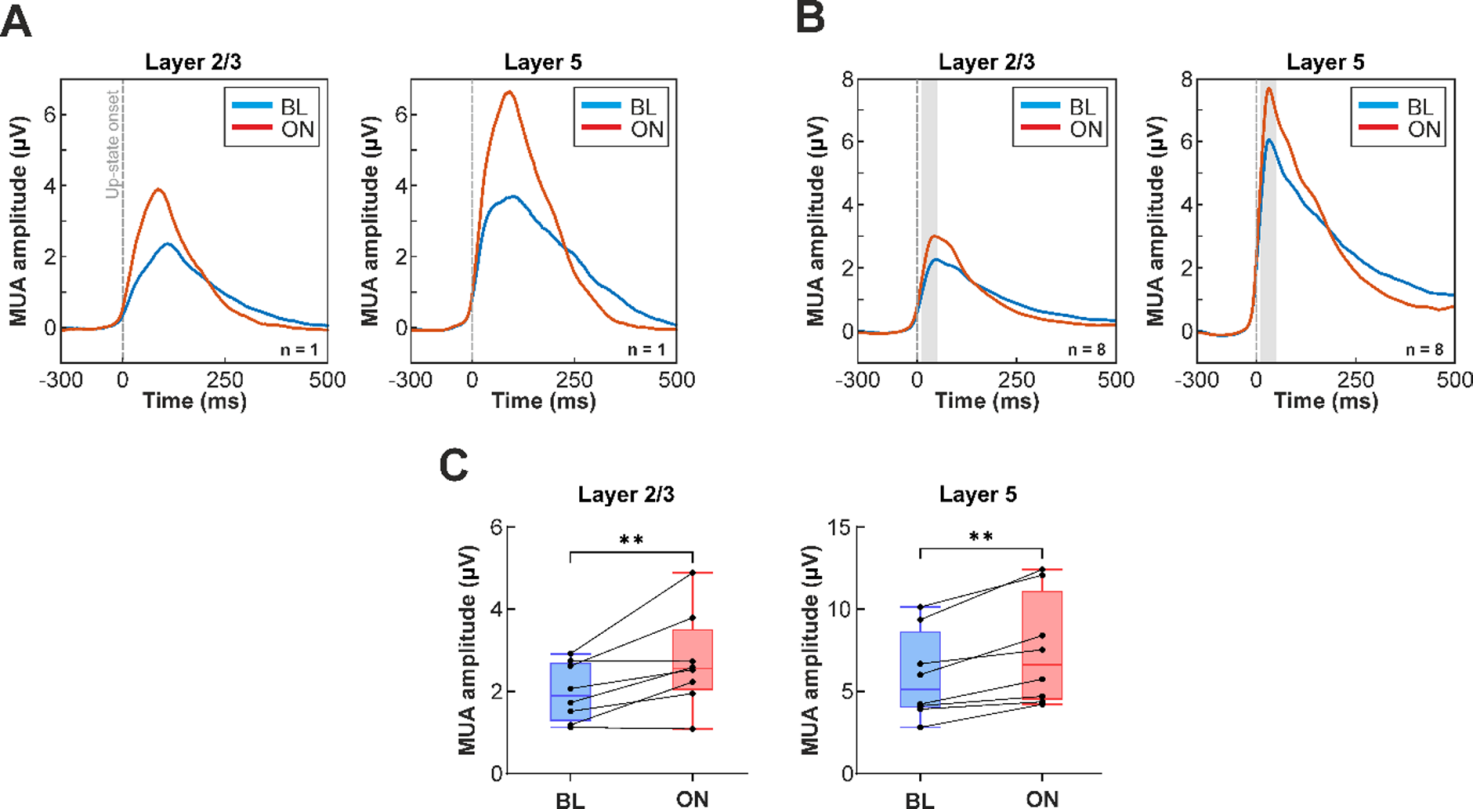
(**A**) Representative examples of multi-unit activity (MUA) traces averaged across up-states from one rat during baseline (BL, blue) and infrared stimulation (ON, red) in layer 2/3 (left) and layer 5 (right). The up-state starts at time point zero (dashed gray vertical line). (**B**) Average of up-state onset locked MUA across all rats (*n* = 8) during baseline (blue) and stimulation (red) in layer 2/3 (left) and layer 5 (right). The shaded gray area indicates the time window used to calculate MUA amplitude values shown in panel C. (**C**) Distribution of mean MUA amplitudes calculated within a 40-ms time window (10–50 ms after up-state onset; see panel B) during baseline and stimulation in layer 2/3 (left) and layer 5 (right). Each dot represents an individual rat; lines connect repeated measures. NIR stimulation significantly increased MUA amplitude across layers, and layer 5 showed higher overall MUA than layer 2/3 (three-way repeated-measures ANOVA: cortical layer F(1,6) = 15.83, *p* = 0.007, ηp²=0.73; NIR stimulation F(1,6) = 22.49, *p* = 0.003, ηp²=0.79; cortical region F(1,6) = 0.09, *p* = 0.78, ηp²=0.014). ** indicates significant main effect of NIR stimulation.

**Fig. 4:**
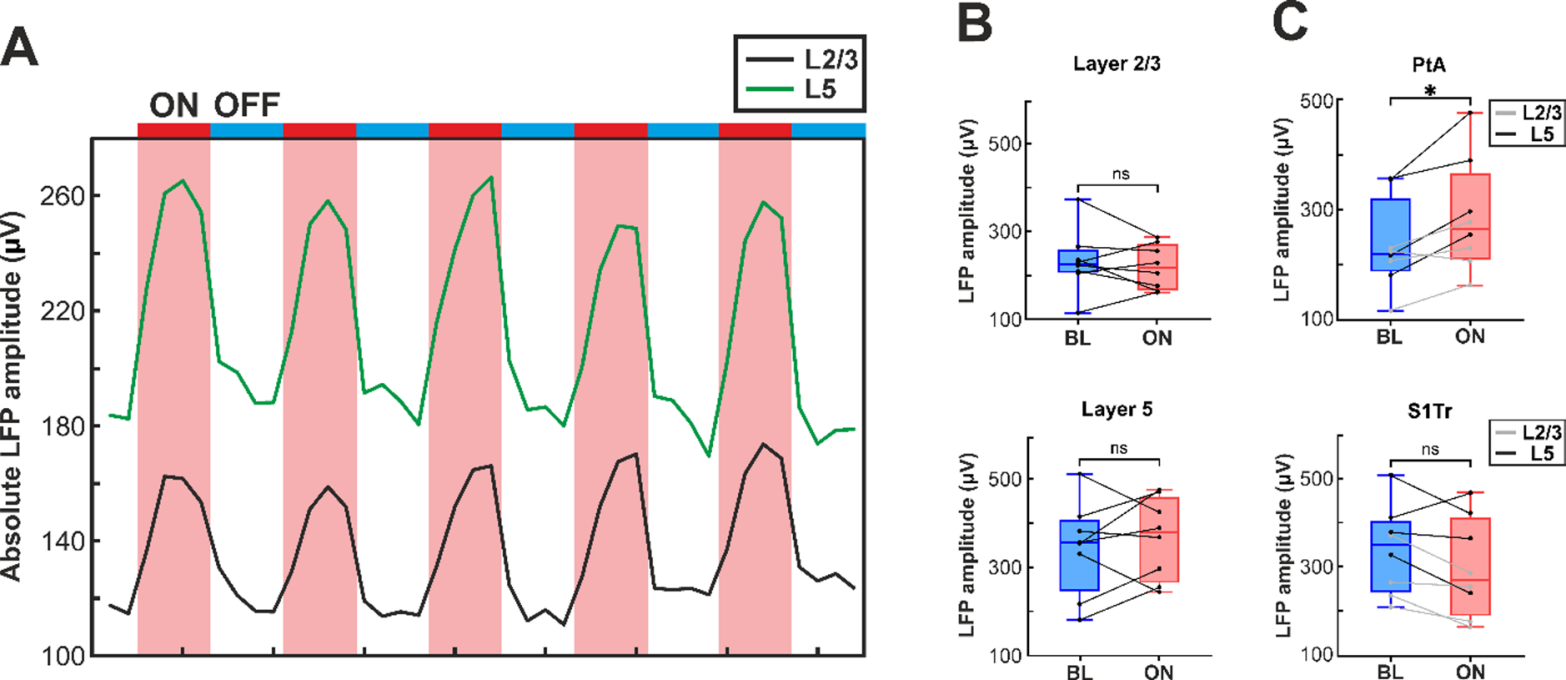
(**A**) Representative example of changes in the absolute LFP amplitude over time in layer 2/3 (L2/3, black) and layer 5 (L5, green) during the infrared stimulation protocol. The red and blue horizontal bars indicate the stimulation (ON) and recovery (OFF) periods, respectively. (**B**) Distribution of mean absolute LFP amplitudes during baseline (BL) and NIR stimulation (ON) in layer 2/3 (top) and layer 5 (bottom) across all rats. Individual data points represent single animals. No significant simple effect of NIR stimulation was observed within either cortical layer. (**C**) Mean absolute LFP amplitudes in the parietal association cortex (PtA, top) and the trunk region of the primary somatosensory cortex (S1Tr, bottom) during BL and ON. NIR stimulation significantly increased LFP amplitudes in PtA (*p* = 0.050, paired t-test), whereas no significant effect was observed in S1Tr. * indicates a significant simple effect of NIR stimulation; ns, not significant.

**Fig. 5:**
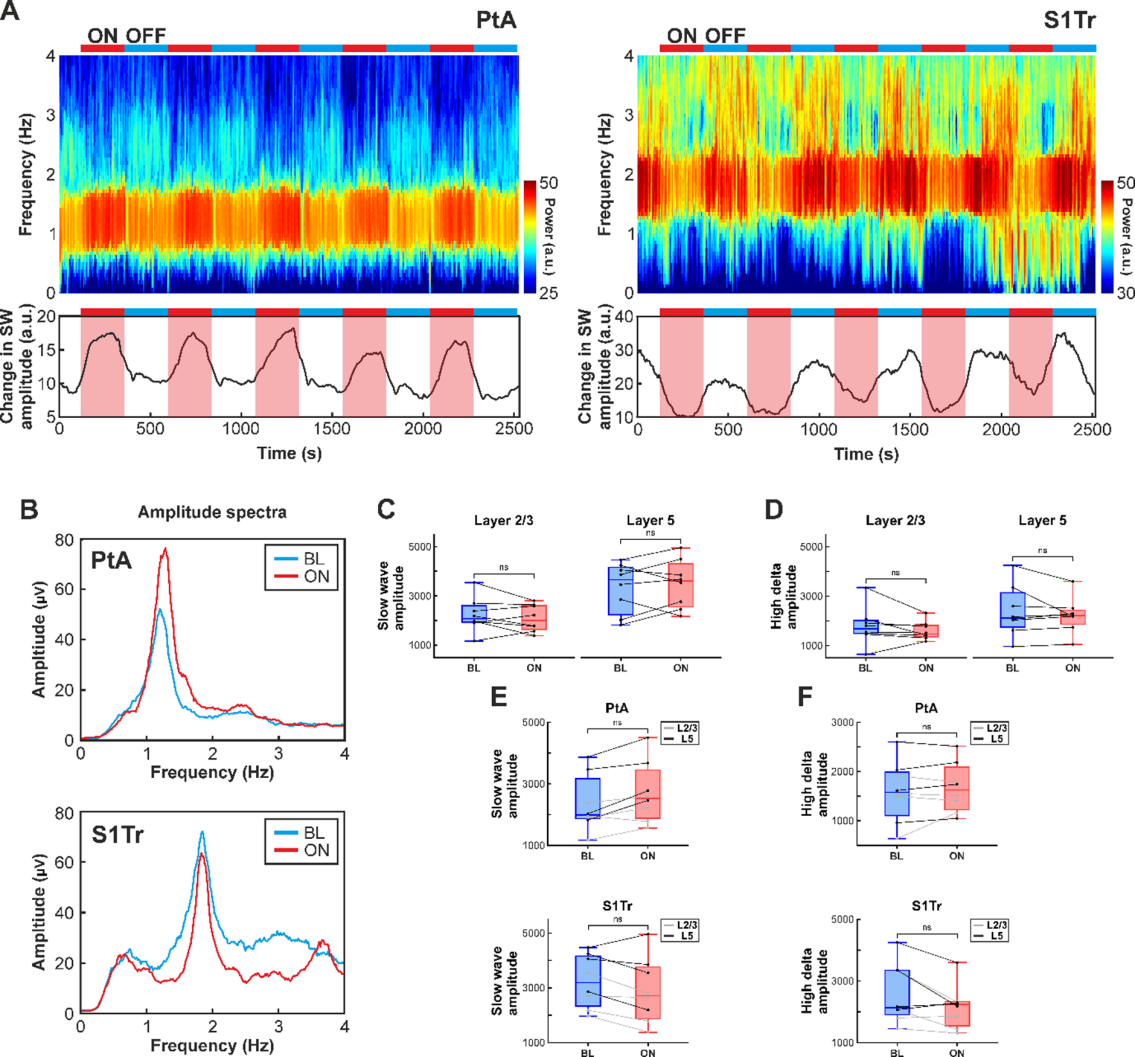
(**A**) Spectrograms computed from layer 5 local field potentials demonstrating spectral changes during the whole infrared stimulation protocol in two rats. In one rat (optrode implanted in PtA, left), the power of low-frequency (0.5–4 Hz) activity increased during stimulation, while in the other rat (optrode implanted in S1Tr, right), a decrease in spectral power occurred during stimulation. The change in the power of the slow wave band (0.5–2 Hz) is shown below the spectrograms. The red and blue horizontal bars indicate the stimulation (ON) and recovery (OFF) periods, respectively. (**B**) Representative FFT amplitude spectra from layer 5 of the same two rats in the low-frequency band (0.5–4 Hz) during baseline (blue) and stimulation (red) periods. (**C**,**D**) Distribution of mean spectral amplitudes in the slow wave (0.5–2 Hz, C) and high delta (2–4 Hz, D) bands during stimulation (ON) compared to baseline (BL) in layer 2/3 (left) and layer 5 (right) across all rats. No significant simple effect of NIR stimulation was observed in either cortical layer. (**E**,**F**) Same as panels C-D, but grouped by cortical region (PtA, top; S1Tr, bottom). No significant simple effects of NIR stimulation were observed in either region for either frequency band. ns, not significant; PtA, parietal association cortex; S1Tr, trunk region of the primary somatosensory cortex.

All electrophysiological data were processed offline with MATLAB (MathWorks, Natick, MA, USA)-based algorithms. Since cortical temperature returns to baseline values within two minutes after cessation of NIR stimulation^54^, we defined the last minute of the four-minute-long OFF period as the baseline (BL) period for the subsequent stimulation trial (Fig. 1B). For the first stimulation trial, the baseline period was the second minute of the cortical recording containing spontaneous activity. All subsequent analyses were applied separately for each minute of the ON period. For the main analyses presented (Figs. 2, 3, 4 and 5), however, we compared values of the baseline period with those from the last minute of the stimulation period, during which the effect of infrared stimulation was typically strongest. To visualize the distribution of state durations (Fig. 2D–E), the last two minutes of ON and OFF periods were used to ensure a sufficient number of up-states and down-states. Computed values were averaged across the five trials and across channels corresponding to the same layer. Using permutation tests, we found no statistically significant differences between the results obtained after 2- and 4-min NIR stimulation; therefore, these data were pooled.

#### Laminar location of recording sites

The tip of the probe was positioned in the infragranular cortical layers, while microelectrodes recorded simultaneously from multiple layers. To determine the precise laminar location of optrode sites, we utilized both the registered cortical activity patterns and, where available, anatomical information (Nissl-stained coronal brain sections containing the optrode track). Each recording site was assigned to a specific cortical layer, based on criteria established in our previous work^4^.

Under ketamine/xylazine anesthesia, superficial layers typically exhibit sparse spontaneous population activity, with fewer neurons active compared to deeper layers^4,14,58^. Due to the absence of spiking activity in layer 1, sites localized to this layer were excluded from further analysis. Recording sites located within layers 2 and 3 were grouped together. Since layer 4 is a thin layer and virtually absent in the parietal association cortex, data from sites potentially located in this layer (in S1Tr) were also excluded.

Layer 5, which contains the cell bodies of the largest pyramidal cells across layers, displays the strongest neuronal activity under ketamine/xylazine anesthesia^4^. This layer also plays a key role in the generation of slow waves^4,10,12,13^. Recording sites in layer 5 were typically used as a reference for identifying other layers, as this layer can be located with the greatest reliability. Finally, since only relatively few sites were located in layer 6, activity from this layer was not analyzed. Thus, the present study focused primarily on neuronal activity in two cortical layers: layer 2/3 and layer 5.

#### Multi-unit activity-based state detection

To examine NIR stimulation-related changes in the duration of up- and down-states and in neuronal population activity (multi-unit activity; MUA, Fig. 2), we first identified the onset of slow oscillation states. The MUA-based state detection algorithm applied here was adapted from Fiáth et al. (2016)^4^. Briefly, MUA was extracted by bandpass filtering the wideband recordings between 500 and 5000 Hz with a third-order, zero-phase shift Butterworth filter. The resulting signal was then rectified and downsampled from 20 kHz to 2 kHz. Downsampled MUA signals from all twelve channels were summed (excluding nonfunctional or noisy channels), and the resulting single-channel time series was then smoothed to obtain the envelope of cortical population activity with a third-order, zero-phase shift Butterworth lowpass filter (50 Hz cutoff frequency). This signal was termed as smoothed population activity (SPA). Finally, to detect up- and down-state onset times, an amplitude threshold was computed based on the mean and standard deviation of short (50 ms) periods of the SPA signal selected within down-states. The detection algorithm imposed two constraints: a minimum up-state duration of 50 ms and a minimum down-state duration of 100 ms. The detected up- and down-state onsets were saved for subsequent analysis.

#### Calculation of up- and down-state durations

Up- and down-state durations were computed using the previously extracted up- and down-state onset times. The duration of a given up-state (or down-state) was defined as the time interval between the onset of the current up-state (or down-state) and the onset of the following down-state (or up-state). To improve reliability, up- and down-states with excessively short or long durations (i.e., exceeding three scaled median absolute deviations from the median), which likely resulted from inaccurate detections, were excluded from the analysis.

#### Computation of up-state onset-locked multi-unit activity averages

To calculate MUA averages aligned to the start of up-states for both the stimulation (ON) and the recovery (OFF) period (Fig. 3), we adapted the method described by Horváth et al. (2024)^53^. Briefly, short segments were extracted from the continuous MUA recordings around each detected up-state onset (from 150 ms before to 500 ms after the onset). For each stimulation trial (separately for ON and OFF periods), these segments were then averaged across up-states and across cortical channels corresponding to either layer 2/3 or to layer 5. Resulting segments were subsequently averaged across trials. Only up-states detected during the last minute of the ON (471.67 ± 118.93 up-states per animal; mean ± standard deviation) and OFF periods (468.63 ± 111.17 up-states per animal) were used to compute the MUA averages. Finally, we calculated the average MUA level within a 40 ms window starting 10 ms after the up-state onset, both during stimulation and the baseline period.

#### Comparison of local field potential amplitudes

We also investigated whether NIR stimulation had a measurable effect on the amplitude of cortical slow waves (Fig. 4). To extract local field potentials, the recorded wideband (0.1–7500 Hz) cortical signals were bandpass filtered between 0.5 and 200 Hz with a third-order, zero-phase shift Butterworth filter. To eliminate the power line noise, a second-order infinite impulse response notch filter was applied at 50 Hz. To reduce data size and computation time, LFP signals were downsampled to 2 kHz. Nonfunctional and noisy channels were removed from further analysis (*n* = 8 channels in total across the 8 recordings).

To assess the influence of NIR stimulation on LFP amplitudes, we calculated and compared the mean absolute LFP amplitude during the last minute of ON and OFF periods, the latter used as baseline. Amplitudes were also averaged across trials and across channels corresponding to the same layer.

#### Spectral analysis of local field potentials

Fast Fourier transformation (FFT) was applied to the extracted LFP signal to calculate the amplitude spectrum in the low-frequency band from 0.5 Hz to 4 Hz (Fig. 5B). Next, we calculated the average spectral amplitude in two separate bands: from 0.5 Hz to 2 Hz, referred to as the slow wave band, and from 2 Hz to 4 Hz, referred to as high delta band. The FFT amplitude spectrum was computed separately for the last minute of the ON and OFF periods, as well as for each channel. Spectral amplitude values were averaged across trials and across channels corresponding to the same cortical layer. To visualize spectral changes during the entire recording, spectrograms were computed using the Chronux MATLAB package^59^(Fig. 5A).

### Statistical analysis

Statistical analyses were performed using GraphPad (v10.3.1) or MATLAB. Normality of the data was assessed using the Shapiro–Wilk and Kolmogorov-Smirnov tests. For up- and down-state durations and slow oscillation peak frequencies, Student’s t-test or paired t-test was used to compare groups. MUA, LFP and spectral amplitudes were analyzed using a three-way mixed-design repeated-measures ANOVA with cortical region (PtA vs. S1Tr) as a between-subjects factor and cortical layer (layer 2/3 vs. layer 5) and NIR stimulation (BL vs. ON) as within-subjects factors. Because all factors had only two levels, significant interactions were followed by analyses of simple effects (e.g., effect of NIR stimulation in each cortical layer or region) without the need for additional post hoc tests. Simple effects were examined using paired t-tests. A p-value < 0.05 was considered statistically significant. Box-and-whisker plots (see Figs. 2, 3, 4 and 5; Supplementary Fig. S1) showing the distribution of data are presented as follows. The middle line indicates the median, the boxes correspond to the 25th and 75th percentiles, and the whiskers mark the minimum and maximum values. Individual values are depicted as black dots. Unless otherwise stated, all values in the Results section are reported as mean ± standard deviation.

## Results

### Infrared stimulation modulates spontaneous cortical slow-wave activity in anesthetized rats

We used a multimodal probe (referred to as an optrode) with its tip inserted into the infragranular layers of the neocortex of ketamine/xylazine anesthetized rats (*n* = 8) to deliver near-infrared light stimulation while simultaneously recording cortical local field potentials and multi-unit activity. The optrode contained twelve, equidistantly spaced microelectrodes, allowing for recordings across multiple cortical layers (Fig. 1A). The device was implanted either in the parietal association cortex (PtA, *n* = 4 rats) or in the trunk region of the primary somatosensory cortex (S1Tr, *n* = 4).

In each animal, continuous-wave NIR stimulation was applied for 4 min (ON period, 2 min in 2 rats), followed by a 4-minute recovery period without stimulation (OFF period; Fig. 1B). Each ON-OFF cycle was repeated five times per animal, with all recordings starting with a two-minute baseline period.

NIR stimulation locally elevates cortical temperature by several degrees Celsius, with the most pronounced increase occurring within 1 mm of the probe tip^46,49,50^. This stimulation-induced hyperthermia led to marked alterations in the properties of ketamine/xylazine-induced cortical slow-wave activity. Changes were observed in both low-frequency (< 200 Hz; LFP) and high-frequency (500–5000 Hz; MUA) components of the recorded neural signal (Fig. 1C). For instance, both the duration of up- and down-states and the amplitudes of slow waves were modulated during stimulation compared to baseline. These effects persisted throughout stimulation and were fully reversible, with parameters rapidly returning to baseline once NIR stimulation ceased. Furthermore, the effects of local cortical warming on slow wave properties were consistent and reproducible across repeated stimulation trials.

To assess potential layer-specific effects, we analyzed stimulation-induced responses separately in supragranular (i.e., layer 2/3) and infragranular (i.e., layer 5) cortical layers (Fig. 1C). In addition, we compared the effects of stimulation between cortical areas (PtA vs. S1Tr).

### Effect of infrared stimulation on the duration of up- and down-states

Cortical slow waves consist of two alternating phases: up-states, characterized by high synaptic activity and neuronal spiking, and down-states, marked by near-complete neuronal silence. Under ketamine/xylazine anesthesia, both phases typically last several hundred milliseconds. Previous studies have demonstrated that the duration of these states is sensitive to tissue temperature^36,37^. We therefore examined whether NIR stimulation alters the duration of up- and down-states.

Up- and down-state onsets were detected using a method based on cortical population activity (Fig. 2A). This approach typically provides precise estimates of state onset times and allows accurate measurement of individual state durations^4^. We found no statistically significant differences in baseline up- and down-state durations between PtA and S1Tr, although up-states were slightly longer in PtA (up-state: PtA, 362.28 ± 86.15 ms; S1Tr, 292.24 ± 114.16 ms, *p* = 0.308, Student’s t-test; down-state: PtA, 329.25 ± 69.95 ms; S1Tr, 340.85 ± 94.06 ms, *p* = 0.826, Student’s t-test).

Visualization of population activity across states detected during stimulation and recovery revealed clear changes in the duration of up- and down-states (Fig. 2B and C). The distribution of state durations also shifted noticeably during stimulation (Fig. 2D and E). Specifically, NIR stimulation significantly reduced up-state duration (BL: 320.89 ± 94.87 ms; ON: 262.40 ± 85.29 ms; *p* = 0.0012, paired t-test; Fig. 2F), while down-states became significantly longer relative to baseline (BL: 337.01 ± 76.29 ms; ON: 384.71 ± 81.16 ms; *p* < 0.0001, paired t-test; Fig. 2G). Consequently, the proportion of time spent in up-states decreased from 48.77 to 37.38%, while down-states increased from 51.23 to 62.62% during stimulation. Despite these changes, the total duration of slow oscillation cycles (the sum of the duration of a single up- and down-state) remained largely unchanged during the whole recording, which was found 662.31 ms and 660.40 ms for the last minute of OFF and ON periods, respectively (Fig. 2H). Changes in state durations progressed gradually during both stimulation and recovery (Fig. 2H).

To examine changes in the distribution of state durations between baseline and stimulation conditions, we calculated the coefficients of variation (CV) for both up- and down-state durations. The CV of up-state durations exhibited a decreasing trend during stimulation, with 7 out of 8 animals showing lower CVs compared to baseline (BL: 0.374 ± 0.073; ON: 0.348 ± 0.081; Supplementary Fig. S1A), although this difference did not reach statistical significance (*p* = 0.080, paired t-test). Similarly, the CV of down-state durations showed a slight decrease during stimulation (BL: 0.305 ± 0.056; ON: 0.285 ± 0.052; Supplementary Fig. S1B), with no significant difference between conditions (*p* = 0.128, paired t-test). Together, these results suggest a weak trend toward reduced variability in state durations, indicating that infrared stimulation may render cortical slow-wave activity slightly more rhythmic, as also illustrated in Fig. 1C.

### Effect of infrared stimulation on population activity

In addition to reducing the duration of up-states, NIR stimulation also increased MUA amplitudes (Fig. 2B). To investigate this effect further, we computed the average MUA aligned to the onset of detected up-states in supragranular (layer 2/3) and infragranular (layer 5) layers, separately for stimulation and recovery periods (Fig. 3A and B).

In most animals, MUA amplitude during up-states was higher during NIR stimulation compared to baseline (Fig. 3A and B). Additionally, the slope of MUA traces was steeper at both down-to-up and up-to-down transitions, indicating sharper and more synchronous state transitions during stimulation.

To quantify the effect of stimulation, we measured the mean MUA amplitude within a 40-ms window (10–50 ms) following the up-state onset during the last minute of the stimulation period and compared it with the corresponding baseline period (last minute of recovery).

A three-way mixed-design repeated-measures ANOVA revealed significant main effects of cortical layer (F(1,6) = 15.83, *p* = 0.007, ηp² = 0.725) and NIR stimulation (F(1,6) = 22.49, *p* = 0.003, ηp² = 0.789) on MUA amplitude, while the main effect of cortical region (PtA vs. S1Tr) was not significant (F(1,6) = 0.09, *p* = 0.78, ηp² = 0.014). No significant two-way or three-way interactions were observed.

Across cortical regions and stimulation conditions, MUA amplitudes were significantly higher in layer 5 compared to layer 2/3 (layer 2/3: 2.36 ± 0.97 µV; layer 5: 6.67 ± 2.92 µV), in agreement with previous findings^4^. NIR stimulation significantly increased MUA amplitude relative to baseline across both layers (layer 2/3: BL, 1.99 ± 0.66 µV; ON, 2.72 ± 1.08 µV; layer 5: BL, 5.91 ± 2.50 µV; ON, 7.43 ± 3.11 µV; Fig. 3C). Together, these results indicate that NIR stimulation enhances MUA amplitude independently of cortical region and layer.

### Effect of infrared stimulation on the amplitude of local field potentials

Changes in the amplitude of slow waves in the LFP signal typically indicate alterations in neural synchronization, with higher amplitudes reflecting greater network-level synchrony of underlying neural activity^60–63^. Given that the increase in MUA amplitude during NIR stimulation suggests enhanced neural synchrony, we next asked whether this effect was also reflected in the amplitude of local field potentials. To assess whether local cortical warming via NIR stimulation modulates the temporal synchrony of spontaneous activity, we calculated mean absolute LFP amplitudes during both the stimulation and recovery periods in supragranular (layer 2/3) and infragranular (layer 5) layers of the neocortex.

A three-way mixed-design repeated-measures ANOVA revealed a significant main effect of cortical layer (F(1,6) = 12.20, *p* = 0.013, ηp² = 0.670), indicating that LFP amplitudes were higher in layer 5 compared to layer 2/3 (layer 2/3: 225.97 ± 60.20 µV; layer 5: 353.93 ± 96.35 µV). The main effects of cortical region (F(1,6) = 2.53, *p* = 0.163, ηp² = 0.297) and NIR stimulation (F(1,6) = 0.098, *p* = 0.765, ηp² = 0.016) were not significant.

However, significant two-way interactions were observed for cortical layer × NIR stimulation (F(1,6) = 11.95, *p* = 0.014, ηp² = 0.666) and cortical region × NIR stimulation (F(1,6) = 9.34, *p* = 0.022, ηp² = 0.609). No other interactions were significant.

To follow up on the layer × stimulation interaction, simple effects of NIR stimulation within each cortical layer were examined using paired t-tests. These analyses revealed no significant effect of NIR stimulation in either layer (layer 2/3: *p* = 0.500; layer 5: *p* = 0.446), indicating that the layer-specific NIR stimulation effect was not statistically significant despite the interaction. Descriptively, NIR stimulation sligthly decreased LFP amplitudes in layer 2/3 and slightly increased amplitudes in layer 5 (layer 2/3: BL, 232.31 ± 71.71 µV; ON, 219.63 ± 50.31 µV; layer 5: BL, 342.85 ± 105.47 µV; ON, 365.00 ± 92.13 µV; Fig. 4B).

Examination of the cortical region × stimulation interaction showed a significant simple effect of NIR stimulation in PtA (*p* = 0.050, paired t-test), with LFP amplitudes increasing during stimulation (BL: 234.96 ± 82.36 µV; ON: 285.98 ± 101.86 µV; Fig. 4C). In contrast, LFP amplitudes in S1Tr slightly decreased during stimulation, although this effect was not significant (*p* = 0.205, paired t-test; BL: 340.20 ± 100.68 µV; ON: 298.65 ± 112.38 µV; Fig. 4D).

These results suggest that NIR stimulation increased LFP amplitude in PtA but not in S1Tr, while layer-dependent differences were present but did not reach significance within individual layers. Overall, mean absolute LFP amplitudes were higher in layer 5 than layer 2/3, and NIR stimulation had a region-specific effect in PtA, reflecting the significant interactions observed in the repeated-measures ANOVA.

### Spectral changes in local field potentials during infrared stimulation

To further examine the effects of NIR stimulation on cortical local field potentials, we analyzed spectral changes in the low-frequency band (0.5–4 Hz) corresponding to ketamine/xylazine-induced slow waves (0.5–2 Hz^4,7^), and high delta activity (2–4 Hz) which represents faster delta oscillations. NIR stimulation, accompanied by local cortical warming, induced notable alterations in the spectral amplitude of low-frequency components (Fig. 5A and B).

Similarly to the results of the LFP amplitude analysis, a subset of animals showed a robust increase in the power of the slow wave band (Fig. 5A, left), while others exhibited a marked decrease during stimulation (Fig. 5A, right). These stimulation-induced spectral changes in the LFP reversed rapidly during the recovery period following the cessation of stimulation (Fig. 5A and B). Trial-to-trial variability in spectral changes was relatively low for most animals (e.g., see Fig. 5A).

To quantify these effects, we calculated the spectral amplitude in the two frequency bands described above (i.e., slow wave and high delta bands) for each animal using the last minute of the stimulation (ON) and the recovery periods (BL; Fig. 5C–F).

For the slow wave band, a three-way mixed-design repeated-measures ANOVA revealed a significant main effect of cortical layer (F(1,6) = 10.87, *p* = 0.016, ηp² = 0.644), indicating that spectral amplitudes in the slow wave band were higher in layer 5 compared to layer 2/3 (layer 2/3: 2163.63 ± 602.92 µV; layer 5: 3423.67 ± 960.45 µV). The main effects of cortical region (F(1,6) = 2.02, *p* = 0.205, ηp² = 0.252) and NIR stimulation (F(1,6) = 0.002, *p* = 0.961, ηp² < 0.001) were not significant.

Significant two-way interactions were observed for cortical layer × NIR stimulation (F(1,6) = 14.16, *p* = 0.009, ηp² = 0.702) and cortical region × NIR stimulation (F(1,6) = 8.97, *p* = 0.024, ηp² = 0.599), while no other interactions were significant.

The layer × stimulation interaction revealed no significant simple effect of NIR stimulation in either layer (layer 2/3: *p* = 0.413; layer 5: *p* = 0.517, paired t-tests; Fig. 5C). Descriptively, NIR stimulation sligthly decreased spectral amplitudes in the slow wave band in layer 2/3 and slightly increased spectral amplitudes in layer 5 (Table 1).

Examination of the cortical region × stimulation interaction showed no significant simple effect of NIR stimulation in either PtA (*p* = 0.056, paired t-test) or the S1Tr (*p* = 0. 187, paired t-test; Fig. 5E). Nevertheless, and consistent with the LFP amplitude results, spectral amplitudes in the slow-wave band showed a trend toward an increase during NIR stimulation in PtA, while a slight decrease was observed in S1Tr (Table 1; Fig. 5E).

For the high delta band (Fig. 5D and F), no significant main effects of cortical region or NIR stimulation were observed, and no significant two-way or three-way interactions were detected (all *p* > 0.10). A trend toward a main effect of cortical layer was observed (F(1,6) = 5.84, *p* = 0.052, ηp² = 0.493), suggesting higher spectral amplitudes in the high delta band in layer 5 compared to layer 2/3, although this difference did not reach statistical significance (layer 2/3: 1691.70 ± 589.30 µV; layer 5: 2340.14 ± 847.51 µV).

Interestingly, in the three rats with S1Tr implantations that showed a decrease in LFP and spectral amplitudes in layer 5 during stimulation (Figs. 4C and 5D), the stimulation site (oprode tip) was likely positioned 200–300 μm more superficially than in the other animals, as indicated by the computed MUA depth profiles (Supplementary Fig. S2). In these rats, based on anatomical and electrophysiological markers, the first recording site (closest to the optrode tip) was localized to layer 5, whereas in the remaining animals the first sites sampled neural activity from the upper part of layer 6 (Supplementary Fig. S2). Together, these observations suggest that the effect of NIR stimulation may be sensitive to the laminar position of the stimulation device within the cortex, implicating layer-specific modulation by stimulation.

Finally, to assess NIR stimulation-induced changes in the frequency of the ketamine/xylazine–induced slow oscillation, we measured the peak oscillation frequency in layer 5. The mean peak frequency was 1.46 ± 0.28 Hz (range: 1.033–1.867 Hz) during baseline and 1.49 ± 0.28 Hz (range: 1.050–1.883 Hz) during stimulation. Peak frequencies did not differ significantly between baseline and stimulation periods (*p* = 0.149; paired t-test), consistent with the similar duration of slow oscillation cycles observed during ON and OFF conditions (~ 660 ms; Fig. 2H). Similarly, slow oscillation peak frequencies did not differ between PtA and S1Tr (*p* = 0.608; Student’s t-test). The measured values closely match the peak frequency reported in our earlier study^4^, which included a larger sample size, and are consistent with frequencies reported by other groups in rodents^7,64,65^.

**Table 1:** Spectral amplitudes (in µV) in the slow wave (0.5–2 Hz) band (mean ± standard deviation). BL, baseline period; ON, infrared stimulation period; PtA, parietal association cortex; S1Tr, trunk region of the primary somatosensory cortex; L2/3, layer 2/3; L5, layer5.

	Layer	BL	ON
All (*n* = 8)	L2/3	2228.82 ± 691.41	2098.44 ± 539.63
	L5	3352.21 ± 1010.75	3495.13 ± 971.30
PtA (*n* = 4)	L2/3 + L5	2326.01 ± 900.00	2696.86 ± 972.41
S1Tr (*n* = 4)	L2/3 + L5	3255.03 ± 957.93	2896.71 ± 1175.13

## Discussion

Slow-wave activity reflects the synchronized behavior of large neuronal populations within the thalamocortical network and exhibits complex spatiotemporal dynamics. In this study, we modulated spontaneously occurring cortical slow-wave activity using near-infrared stimulation in rats anesthetized with ketamine/xylazine. We observed significant changes in multiple properties of slow waves during stimulation, including their amplitudes, the duration of active and silent states and the synchronization level of population spiking activity.

Interestingly, infrared stimulation-induced changes in the LFP (particularly spectral amplitude and slow wave amplitude) varied across the two investigated cortical areas. In the parietal association cortex, NIR neuromodulation enhanced slow and delta oscillations, manifesting as increased spectral amplitude and larger slow wave amplitudes. In contrast, the primary somatosensory cortex showed a reduction in these measures. Several factors may contribute to this interareal discrepancy.

First, anatomical differences between cortical areas may play a role. For instance, the association cortex lacks a well-defined layer 4 and has a reduced overall cortical thickness compared to primary sensory regions. Consequently, although the optrode tip (i.e., the site of stimulation) was consistently positioned 1.2 mm below the cortical surface, its laminar location varied between cortical regions and animals (see Supplementary Fig. S2). Based on histological analysis, in PtA, the tip was typically located in the upper to middle part of layer 6, while in S1Tr it was in the lower part of layer 5 in most cases (3 out of 4 animals; Supplementary Fig. S2). These laminar differences may have affected the spatial distribution and intensity of temperature changes induced by infrared stimulation.

Second, the size of the craniotomy may have influenced the baseline temperature of the cortical tissue near the optrode. In PtA recordings, the craniotomy (10 × 5 mm^2^) was more than five times larger than in S1Tr recordings (3 × 3 mm^2^). Prior work has demonstrated that exposed cortical tissue in a craniotomy is significantly cooler than physiological brain temperature due to heat loss^36^. In mice, the exposed cortical surface can be up to 10 °C below core body temperature, markedly affecting ongoing cortical activity^36^. A larger craniotomy would thus produce greater heat dissipation, possibly lowering baseline cortical temperature more and perturbing baseline neural activity at a higher degree and on a larger spatial scale. This factor may partially explain the region-specific effects of IR stimulation observed here.

The observed increase in slow/delta amplitude in PtA is consistent with previous findings. For instance, in ferret coronal brain slices from the primary visual cortex expressing slow rhythms in vitro, higher chamber temperatures were associated with increased spectral power in low LFP frequencies^37^. Similarly, Sheroziya and Timofeev (2015)^38^ reported elevated delta band (0.2–4 Hz) power in the motor cortex of head-fixed, non-anesthetized mice at physiological cortical temperatures compared to cooled tissue. Interestingly, contrary to our results in S1Tr, in ketamine/xylazine anesthetized mice, the same study found that higher tissue temperatures in the somatosensory cortex were associated with larger slow wave amplitudes^38^. Kalmbach and Waters (2012)^36^ also reported increased power in the 0–1.5 Hz range at physiological brain temperatures in the barrel cortex of mice anesthetized with isoflurane compared to those measured in cranial windows with lower cortical temperatures.

In contrast to the regional variability of LFP-related measures, other parameters, namely state durations and MUA amplitude, showed consistent changes across animals. Specifically, infrared stimulation shortened up-states and prolonged down-states, accompanied by increased MUA amplitudes and steeper MUA slopes in both supragranular and infragranular layers.

Our findings regarding state durations align partially with above studies. Higher cortical temperatures have been associated with shorter up-states in both the barrel cortex of anesthetized mice and visual cortical slices^36,37^. However, the prolongation of down-states observed in our study during infrared stimulation was only reported by Kalmbach and Waters (2012)^36^. In brain slices, the duration of down-states initially decreased with temperature increases from 32 °C to 36 °C, but then began to lengthen above 38 °C.

In anesthetized rats, infrared stimulation also produced a steeper rise in cortical MUA and higher peak amplitudes during up-states. These findings are consistent with those from brain slice experiments, where warmer chamber temperatures led to sharper population activity during state transitions^37^, potentially reflecting more synchronized neuronal firing with a faster recruitment of local neuronal populations.

Our previous study using a similar optrode device showed that infrared stimulation enhanced low-frequency (1–3 Hz) LFP power and increased MUA amplitude in the neocortex of anesthetized rats^52^. The optrode in this report featured a micromirror tip that allowed for spatially focused delivery of infrared light focused to one side of the silicon shaft. The study found significantly higher delta power and MUA amplitude within 300 μm in front of the optrode shaft, where heating was maximal, compared to the rear side of the device^53^. In some of these experiments, decreased spectral and MUA amplitudes were observed in backside regions less affected by infrared stimulation compared to baseline values. These findings suggest that the spatial reach of infrared stimulation-induced neuromodulation is relatively localized, likely limited to a few hundred micrometers from the optrode tip.

Since the optrode applied in this study contained relatively few and relatively large microelectrodes, yielding only a small number of well-isolated single-units, single-unit analysis was not performed here. Nevertheless, recent studies in rats have investigated the influence of infrared stimulation at the level of single neurons, reporting both excitatory and suppressive effects on firing rates^46,49,50,53^. Under continuous-wave stimulation, as applied here, roughly one-third of identified units exhibited reduced firing rates (~ 45% decrease on average compared to baseline), while approximately one-quarter showed increased firing (~ 110% mean increase in firing rates) in anesthetized rats^46^. Despite this bidirectional modulation, the overall effect of these changes was a modest net increase in neuronal firing, which likely accounts for the enhanced population activity (MUA) observed in our study. Moreover, because infrared stimulation shortened up-states, spikes became confined to briefer periods, producing sharper, higher-amplitude MUA peaks with steeper slopes. This finding further supports the conclusion that infrared stimulation promotes more synchronized slow-wave activity.

In this study, we focused on the effects of infrared stimulation on the activity of neural circuits. However, other cortical cell types, particularly glial cells, may also contribute to the observed changes. For instance, calcium imaging combined with LFP recordings has shown that astrocytes play a role in the generation of slow-wave activity in the primary visual cortex of ketamine/xylazine anesthetized rats^66^. Furthermore, calcium imaging in the somatosensory cortex of urethane-anesthetized rats revealed that pulsed infrared light elicits both slow and fast responses, with the slow component originating from astrocytes^48^. More recently, a comprehensive study using calcium imaging, pharmacology, electrophysiology, and genetic manipulation in rat cortical astrocyte cultures provided detailed insight into how pulsed infrared light modulates astrocytic activity^67^. Taken together, these findings suggest that astrocytes may also contribute to the NIR stimulation-related modulation of cortical slow wave dynamics observed in our study.

## Supplementary Information

Below is the link to the electronic supplementary material.

Supplementary Material 1

## Data Availability

The datasets generated during and/or analysed during the current study are available from the corresponding author on reasonable request.

## References

[1] Achermann, P. & Borbély, A. A. Low-frequency (< 1 Hz) oscillations in the human sleep electroencephalogram. *Neuroscience* **81**, 213–222. 10.1016/s0306-4522(97)00186-3 (1997). 9300413 10.1016/s0306-4522(97)00186-3

[2] Crunelli, V. & Hughes, S. W. The slow (< 1 Hz) rhythm of non-REM sleep: a dialogue between three Cardinal oscillators. *Nat. Neurosci.* **13**, 9–17. 10.1038/nn.2445 (2010). 19966841 10.1038/nn.2445 PMC2980822

[3] Neske, G. T. The slow Oscillation in cortical and thalamic networks: mechanisms and functions. *Front. Neural Circuits*. **9**, 88. 10.3389/fncir.2015.00088 (2016). 26834569 10.3389/fncir.2015.00088 PMC4712264

[4] Fiáth, R. et al. Laminar analysis of the slow wave activity in the somatosensory cortex of anesthetized rats. *Eur. J. Neurosci.* **44**, 1935–1951. 10.1111/ejn.13274 (2016). 27177594 10.1111/ejn.13274

[5] Steriade, M., Nunez, A. & Amzica, F. A novel slow (< 1 Hz) Oscillation of neocortical neurons in vivo: depolarizing and hyperpolarizing components. *J. Neurosci.* **13**, 3252–3265. 10.1523/JNEUROSCI.13-08-03252.1993 (1993). 8340806 10.1523/JNEUROSCI.13-08-03252.1993 PMC6576541

[6] Ward-Flanagan, R., Lo, A. S., Clement, E. A. & Dickson, C. T. A comparison of brain-state dynamics across common anesthetic agents in male Sprague-Dawley rats. *Int. J. Mol. Sci.* **23**, 3608. 10.3390/ijms23073608 (2022). 35408973 10.3390/ijms23073608 PMC8998244

[7] Sharma, A. V., Wolansky, T. & Dickson, C. T. A comparison of sleeplike slow oscillations in the hippocampus under ketamine and urethane anesthesia. *J. Neurophysiol.* **104**, 932–939. 10.1152/jn.01065.2009 (2010). 20538775 10.1152/jn.01065.2009

[8] Clement, E. A. et al. Cyclic and sleep-like spontaneous alternations of brain state under urethane anaesthesia. *PLoS One* 3, e (2004). 10.1371/journal.pone.0002004 (2008). 10.1371/journal.pone.0002004 PMC2289875 18414674

[9] Timofeev, I., Grenier, F., Bazhenov, M., Sejnowski, T. J. & Steriade, M. Origin of slow cortical oscillations in deafferented cortical slabs. *Cereb. Cortex*. **10**, 1185–1199. 10.1093/cercor/10.12.1185 (2000). 11073868 10.1093/cercor/10.12.1185

[10] Sanchez-Vives, M. V. & McCormick, D. A. Cellular and network mechanisms of rhythmic recurrent activity in neocortex. *Nat. Neurosci.* **3**, 1027–1034. 10.1038/79848 (2000). 11017176 10.1038/79848

[11] Beltramo, R. et al. Layer-specific excitatory circuits differentially control recurrent network dynamics in the neocortex. *Nat. Neurosci.* **16**, 227–234. 10.1038/nn.3306 (2013). 23313909 10.1038/nn.3306

[12] Chauvette, S., Volgushev, M. & Timofeev, I. Origin of active States in local neocortical networks during slow sleep Oscillation. *Cereb. Cortex*. **20**, 2660–2674. 10.1093/cercor/bhq009 (2010). 20200108 10.1093/cercor/bhq009 PMC2951844

[13] Lőrincz, M. et al. A distinct class of slow (~ 0.2–2 Hz) intrinsically bursting layer 5 pyramidal neurons determines UP/DOWN state dynamics in the neocortex. *J. Neurosci.* **35**, 5442–5458. 10.1523/JNEUROSCI.3603-14.2015 (2015). 25855163 10.1523/JNEUROSCI.3603-14.2015 PMC4388913

[14] Sakata, S. & Harris, K. D. Laminar structure of spontaneous and sensory-evoked population activity in auditory cortex. *Neuron* **64**, 404–418. 10.1016/j.neuron.2009.09.020 (2009). 19914188 10.1016/j.neuron.2009.09.020 PMC2778614

[15] Wester, J. C. & Contreras, D. Columnar interactions determine horizontal propagation of recurrent network activity in neocortex. *J. Neurosci.* **32**, 5454–5471. 10.1523/JNEUROSCI.5006-11.2012 (2012). 22514308 10.1523/JNEUROSCI.5006-11.2012 PMC3415278

[16] Dasilva, M. et al. Modulation of cortical slow oscillations and complexity across anesthesia levels. *Neuroimage* **224**, 117415. 10.1016/j.neuroimage.2020.117415 (2021). 33011419 10.1016/j.neuroimage.2020.117415

[17] Greenberg, A. & Dickson, C. T. Spontaneous and electrically modulated Spatiotemporal dynamics of the neocortical slow Oscillation and associated local fast activity. *Neuroimage* **83**, 782–794. 10.1016/j.neuroimage.2013.07.034 (2013). 23876244 10.1016/j.neuroimage.2013.07.034

[18] Hangya, B. et al. Complex propagation patterns characterize human cortical activity during slow-wave sleep. *J. Neurosci.* **31**, 8770–8779. 10.1523/JNEUROSCI.1498-11.2011 (2011). 21677161 10.1523/JNEUROSCI.1498-11.2011 PMC3145488

[19] Massimini, M., Huber, R., Ferrarelli, F., Hill, S. & Tononi, G. The sleep slow Oscillation as a traveling wave. *J. Neurosci.* **24**, 6862–6870. 10.1523/JNEUROSCI.1318-04.2004 (2004). 15295020 10.1523/JNEUROSCI.1318-04.2004 PMC6729597

[20] Sheroziya, M. & Timofeev, I. Global intracellular slow-wave dynamics of the thalamocortical system. *J. Neurosci.* **34**, 8875–8893. 10.1523/JNEUROSCI.4460-13.2014 (2014). 24966387 10.1523/JNEUROSCI.4460-13.2014 PMC4069359

[21] Horváth, C., Ulbert, I. & Fiáth, R. Propagating population activity patterns during spontaneous slow waves in the thalamus of rodents. *Neuroimage* **285**, 120484. 10.1016/j.neuroimage.2023.120484 (2024). 38061688 10.1016/j.neuroimage.2023.120484

[22] Born, J., Rasch, B. & Gais, S. Sleep to remember. *Neuroscientist* **12**, 410–424. 10.1177/1073858406292647 (2006). 16957003 10.1177/1073858406292647

[23] Tononi, G. & Cirelli, C. Sleep function and synaptic homeostasis. *Sleep. Med. Rev.* **10**, 49–62. 10.1016/j.smrv.2005.05.002 (2006). 16376591 10.1016/j.smrv.2005.05.002

[24] Tononi, G. & Cirelli, C. Sleep and the price of plasticity: from synaptic and cellular homeostasis to memory consolidation and integration. *Neuron* **81**, 12–34. 10.1016/j.neuron.2013.12.025 (2014). 24411729 10.1016/j.neuron.2013.12.025 PMC3921176

[25] Rasch, B. & Born, J. About sleep’s role in memory. *Physiol. Rev.* **93**, 681–766. 10.1152/physrev.00032.2012 (2013). 23589831 10.1152/physrev.00032.2012 PMC3768102

[26] Timofeev, I. & Chauvette, S. Sleep slow Oscillation and plasticity. *Curr. Opin. Neurobiol.* **44**, 116–126. 10.1016/j.conb.2017.03.019 (2017). 28453998 10.1016/j.conb.2017.03.019

[27] Hablitz, L. M. et al. Increased glymphatic influx is correlated with high EEG delta power and low heart rate in mice under anesthesia. *Sci. Adv.* **5**, eaav5447. 10.1126/sciadv.aav5447 (2019). 30820460 10.1126/sciadv.aav5447 PMC6392807

[28] Xie, L. et al. Sleep drives metabolite clearance from the adult brain. *Science* **342**, 373–377. 10.1126/science.1241224 (2013). 24136970 10.1126/science.1241224 PMC3880190

[29] D’Andola, M. et al. Bistability, causality, and complexity in cortical networks: an in vitro perturbational study. *Cereb. Cortex*. **28**, 2233–2242. 10.1093/cercor/bhx122 (2018). 28525544 10.1093/cercor/bhx122

[30] Hasenstaub, A., Sachdev, R. N. & McCormick, D. A. State changes rapidly modulate cortical neuronal responsiveness. *J. Neurosci.* **27**, 9607–9622. 10.1523/JNEUROSCI.2184-07.2007 (2007). 17804621 10.1523/JNEUROSCI.2184-07.2007 PMC6672966

[31] Kuki, T. et al. Frequency-dependent entrainment of neocortical slow Oscillation to repeated optogenetic stimulation in the anesthetized rat. *Neurosci. Res.* **75**, 35–45. 10.1016/j.neures.2012.10.007 (2013). 23154073 10.1016/j.neures.2012.10.007

[32] Massimini, M. et al. Triggering sleep slow waves by transcranial magnetic stimulation. *Proc. Natl. Acad. Sci. USA*. **104**, 8496–8501. 10.1073/pnas.0702495104 (2007). 17483481 10.1073/pnas.0702495104 PMC1895978

[33] Shu, Y., Hasenstaub, A. & McCormick, D. A. Turning on and off recurrent balanced cortical activity. *Nature* **423**, 288–293. 10.1038/nature01616 (2003). 12748642 10.1038/nature01616

[34] Stroh, A. et al. Making waves: initiation and propagation of corticothalamic Ca²⁺ waves in vivo. *Neuron* **77**, 1136–1150. 10.1016/j.neuron.2013.01.031 (2013). 23522048 10.1016/j.neuron.2013.01.031

[35] Vyazovskiy, V. V., Faraguna, U., Cirelli, C. & Tononi, G. Triggering slow waves during NREM sleep in the rat by intracortical electrical stimulation: effects of sleep/wake history and background activity. *J. Neurophysiol.* **101**, 1921–1931. 10.1152/jn.91157.2008 (2009). 19164101 10.1152/jn.91157.2008 PMC2695630

[36] Kalmbach, A. S. & Waters, J. Brain surface temperature under a craniotomy. *J. Neurophysiol.* **108**, 3138–3146. 10.1152/jn.00557.2012 (2012). 22972953 10.1152/jn.00557.2012 PMC3544864

[37] Reig, R., Mattia, M., Compte, A., Belmonte, C. & Sanchez-Vives, M. V. Temperature modulation of slow and fast cortical rhythms. *J. Neurophysiol.* **103**, 1253–1261. 10.1152/jn.00890.2009 (2010). 20032235 10.1152/jn.00890.2009

[38] Sheroziya, M. & Timofeev, I. Moderate cortical cooling eliminates thalamocortical silent States during slow Oscillation. *J. Neurosci.* **35**, 13006–13019. 10.1523/JNEUROSCI.1359-15.2015 (2015). 26400932 10.1523/JNEUROSCI.1359-15.2015 PMC6605433

[39] Nir, Y. et al. Regional slow waves and spindles in human sleep. *Neuron* **70**, 153–169. 10.1016/j.neuron.2011.02.043 (2011). 21482364 10.1016/j.neuron.2011.02.043 PMC3108825

[40] Siclari, F. & Tononi, G. Local aspects of sleep and wakefulness. *Curr. Opin. Neurobiol.* **44**, 222–227. 10.1016/j.conb.2017.05.008 (2017). 28575720 10.1016/j.conb.2017.05.008 PMC6445546

[41] Funk, C. M., Honjoh, S., Rodriguez, A. V., Cirelli, C. & Tononi, G. Local slow waves in superficial layers of primary cortical areas during REM sleep. *Curr. Biol.* **26**, 396–403. 10.1016/j.cub.2015.11.062 (2016). 26804554 10.1016/j.cub.2015.11.062 PMC4747819

[42] Vyazovskiy, V. V. et al. Local sleep in awake rats. *Nature* **472**, 443–447. 10.1038/nature10009 (2011). 21525926 10.1038/nature10009 PMC3085007

[43] Fekete, Z., Horváth, Á. C. & Zátonyi, A. Infrared neuromodulation: a neuroengineering perspective. *J. Neural Eng.* **17**, 051003. 10.1088/1741-2552/abb3b2 (2020). 33055373 10.1088/1741-2552/abb3b2

[44] Wang, X. et al. Transcranial photobiomodulation with 1064-nm laser modulates brain electroencephalogram rhythms. *Neurophotonics* **6**, 025013. 10.1117/1.NPh.6.2.025013 (2019). 31259198 10.1117/1.NPh.6.2.025013 PMC6563945

[45] Zomorrodi, R., Loheswaran, G., Pushparaj, A. & Lim, L. Pulsed near infrared transcranial and intranasal photobiomodulation significantly modulates neural oscillations: a pilot exploratory study. *Sci. Rep.* **9**, 6309. 10.1038/s41598-019-42693-x (2019). 31004126 10.1038/s41598-019-42693-x PMC6474892

[46] Balogh-Lantos, Z., Fiáth, R., Horváth, Á. C. & Fekete, Z. High density laminar recordings reveal cell type and layer specific responses to infrared neural stimulation in the rat neocortex. *Sci. Rep.* **14**, 31523. 10.1038/s41598-024-82980-w (2024). 39732850 10.1038/s41598-024-82980-w PMC11682324

[47] Cayce, J. M., Friedman, R. M., Jansen, E. D., Mahavaden-Jansen, A. & Roe, A. W. Pulsed infrared light alters neural activity in rat somatosensory cortex in vivo. *Neuroimage* **57**, 155–166. 10.1016/j.neuroimage.2011.03.084 (2011). 21513806 10.1016/j.neuroimage.2011.03.084 PMC3108823

[48] Cayce, J. M. et al. Calcium imaging of infrared-stimulated activity in rodent brain. *Cell. Calcium*. **55**, 183–190. 10.1016/j.ceca.2014.01.004 (2014). 24674600 10.1016/j.ceca.2014.01.004 PMC4014070

[49] Horváth, Á. C. et al. Infrared neural stimulation and Inhibition using an implantable silicon photonic microdevice. *Microsyst. Nanoeng*. **6**, 44. 10.1038/s41378-020-0153-3 (2020). 34567656 10.1038/s41378-020-0153-3 PMC8433474

[50] Horváth, Á. C. et al. Histological and electrophysiological evidence on the safe operation of a sharp-tip multimodal optrode during infrared neuromodulation of the rat cortex. *Sci. Rep.* **12**, 11434. 10.1038/s41598-022-15367-4 (2022). 35794160 10.1038/s41598-022-15367-4 PMC9259743

[51] Horváth, Á. C. et al. A multimodal microtool for spatially controlled infrared neural stimulation in the deep brain tissue. *Sens. Actuators B Chem.* **263**, 77–86. 10.1016/j.snb.2018.02.034 (2018).

[52] Soltani, S. et al. Sleep-Wake cycle in young and older mice. *Front. Syst. Neurosci.* **13**, 51. 10.3389/fnsys.2019.00051 (2019). 31611779 10.3389/fnsys.2019.00051 PMC6769075

[53] Horváth, Á. C. et al. Silicon optrode with a micromirror-tip providing tunable beam profile during infrared neuromodulation of the rat neocortex. *Adv. Mater. Technol.* **9**, 2400044. 10.1002/admt.202400044 (2024).

[54] Ismaiel, E., Fiáth, R., Szabó, Á., Horváth, Á. C. & Fekete, Z. Thermal neuromodulation using pulsed and continuous infrared illumination in a penicillin-induced acute epilepsy model. *Sci. Rep.* **13**, 14460. 10.1038/s41598-023-41552-0 (2023). 37660232 10.1038/s41598-023-41552-0 PMC10475096

[55] Paxinos, G. & Watson, C. *The Rat Brain in Stereotaxic Coordinates: Hard Cover Edition* (Elsevier, 2006).

[56] Csernyus, B. et al. A multimodal, implantable sensor array and measurement system to investigate the suppression of focal epileptic seizure using hypothermia. *J. Neural Eng.* **18**, 0460c3. 10.1088/1741-2552/ac15e6 (2021). 10.1088/1741-2552/ac15e6 34280911

[57] Fiáth, R. et al. Slow insertion of silicon probes improves the quality of acute neuronal recordings. *Sci. Rep.* **9**, 111. 10.1038/s41598-018-36816-z (2019). 30643182 10.1038/s41598-018-36816-z PMC6331571

[58] Barth, A. L. & Poulet, J. F. Experimental evidence for sparse firing in the neocortex. *Trends Neurosci.* **35**, 345–355. 10.1016/j.tins.2012.03.008 (2012). 22579264 10.1016/j.tins.2012.03.008

[59] Bokil, H., Andrews, P., Kulkarni, J. E., Mehta, S. & Mitra, P. P. Chronux: a platform for analyzing neural signals. *J. Neurosci. Methods*. **192**, 146–151. 10.1016/j.jneumeth.2010.06.020 (2010). 20637804 10.1016/j.jneumeth.2010.06.020 PMC2934871

[60] Bernardi, G., Siclari, F., Handjaras, G., Riedner, B. A. & Tononi, G. Local and widespread slow waves in stable NREM sleep: evidence for distinct regulation mechanisms. *Front. Hum. Neurosci.* **12**, 248. 10.3389/fnhum.2018.00248 (2018). 29970995 10.3389/fnhum.2018.00248 PMC6018150

[61] Esser, S. K., Hill, S. L. & Tononi, G. Sleep homeostasis and cortical synchronization: I. Modeling the effects of synaptic strength on sleep slow waves. *Sleep* **30**, 1617–1630. 10.1093/sleep/30.12.1617 (2007). 18246972 10.1093/sleep/30.12.1617 PMC2276134

[62] Riedner, B. A. et al. Sleep homeostasis and cortical synchronization: III. A high-density EEG study of sleep slow waves in humans. *Sleep* **30**, 1643–1657. 10.1093/sleep/30.12.1643 (2007). 18246974 10.1093/sleep/30.12.1643 PMC2276133

[63] Vyazovskiy, V. V., Riedner, B. A., Cirelli, C. & Tononi, G. Sleep homeostasis and cortical synchronization: II. A local field potential study of sleep slow waves in the rat. *Sleep* **30**, 1631–1642. 10.1093/sleep/30.12.1631 (2007). 18246973 10.1093/sleep/30.12.1631 PMC2276140

[64] David, F. et al. Essential thalamic contribution to slow waves of natural sleep. *J. Neurosci.* **33**, 19599–19610. 10.1523/JNEUROSCI.3169-13.2013 (2013). 24336724 10.1523/JNEUROSCI.3169-13.2013 PMC3858629

[65] Dworak, M., McCarley, R. W., Kim, T. & Basheer, R. Delta oscillations induced by ketamine increase energy levels in sleep-wake related brain regions. *Neuroscience* **197**, 72–79. 10.1016/j.neuroscience.2011.09.027 (2011). 21958867 10.1016/j.neuroscience.2011.09.027 PMC3576049

[66] Szabó, Z. et al. Extensive astrocyte synchronization advances neuronal coupling in slow wave activity in vivo. *Sci. Rep.* **7**, 6018. 10.1038/s41598-017-06073-7 (2017). 28729692 10.1038/s41598-017-06073-7 PMC5519671

[67] Borrachero-Conejo, A. I. et al. Stimulation of water and calcium dynamics in astrocytes with pulsed infrared light. *FASEB J.* **34**, 6539–6553. 10.1096/fj.201903049R (2020). 32202681 10.1096/fj.201903049R

