# Supplementary material for: Local infrared stimulation modulates spontaneous cortical slow wave dynamics in anesthetized rats
Source: Sci Rep. 2026 Feb 5;16:7446. doi: 10.1038/s41598-026-38781-4 (PMC12929577; doi:10.1038/s41598-026-38781-4)
Supplement: Supplementary file 1 — Supplementary Material 1 [file 41598_2026_38781_MOESM1_ESM.docx]

**Supplementary Material**

Local infrared stimulation modulates spontaneous cortical slow wave dynamics in anesthetized rats

**Ágnes Szabó^1,+^, Richárd Fiáth^1,2,+^, Ágoston Csaba Horváth^1^, Péter Barthó^2^, István Ulbert^1,2,3,^* and Zoltán Fekete^1,2,†^**

^1^ Faculty of Information Technology and Bionics, Pázmány Péter Catholic University, Práter utca 50/A, 1083 Budapest, Hungary

^2^ Institute of Cognitive Neuroscience and Psychology, HUN-REN Research Centre for Natural Sciences, Magyar tudósok körútja 2., 1117 Budapest, Hungary

^3^ Department of Neurosurgery and Neurointervention, Faculty of Medicine, Semmelweis University, Amerikai út 57., 1145 Budapest, Hungary

* Corresponding author: István Ulbert, Institute of Cognitive Neuroscience and Psychology, HUN-REN Research Centre for Natural Sciences, Magyar tudósok körútja 2., 1117 Budapest, Hungary

Email: [ulbert.istvan@ttk.hu](mailto:ulbert.istvan@ttk.hu)

**^+^** These authors contributed equally to this work.


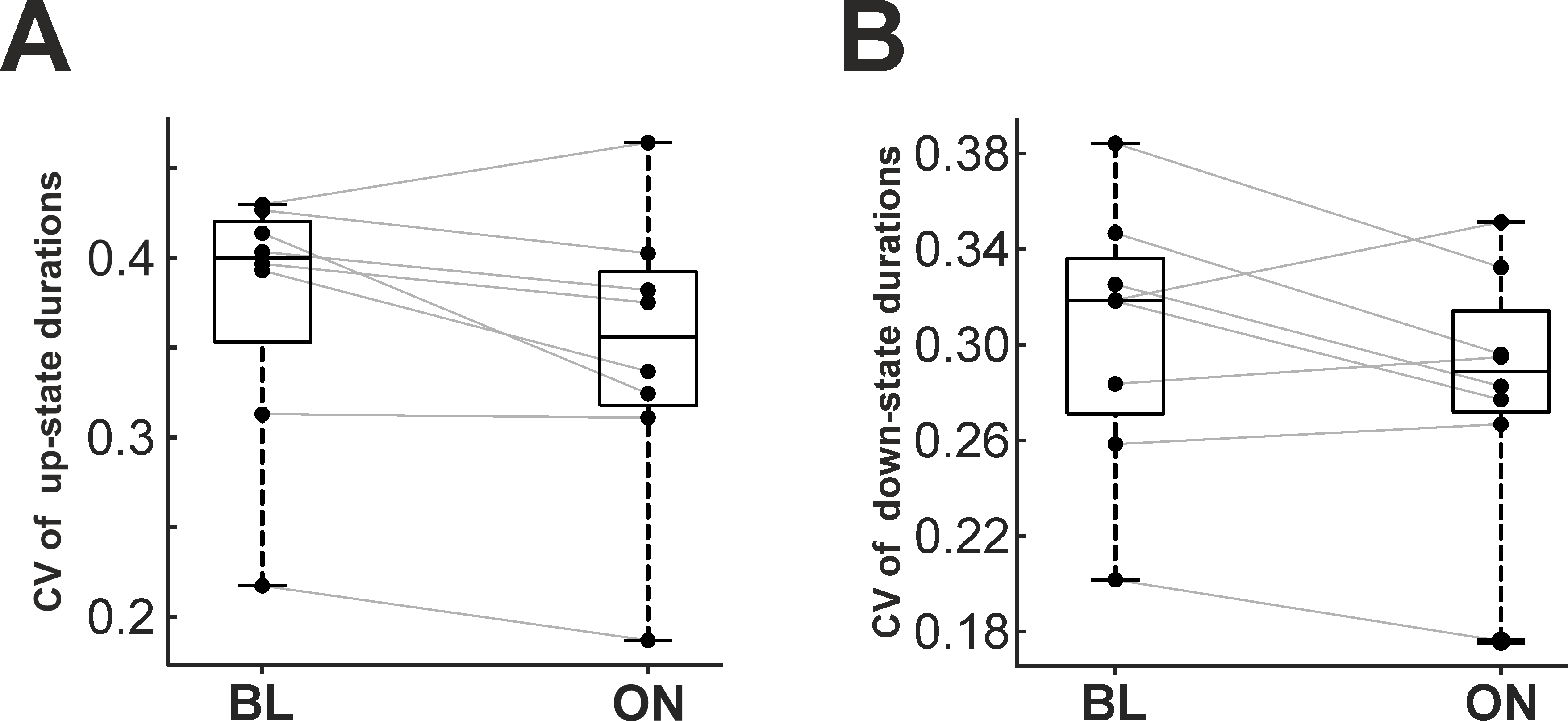


**Supplementary Figure S1.** Variability of up-state (A) and down-state (B) durations during baseline (BL) and stimulation (ON) periods, quantified by the coefficients of variation (CV).





**Supplementary Figure S2.** Amplitude spectra between 1-4 Hz for all rats (Rat 1-8) with PtA (A; Rat 1-4) and S1Tr (B, Rat 5-8) implantations during baseline (BL, blue) and stimulation (ON, red) periods. Both for (A) and (B), the bottom rows show the laminar multi-unit activity (MUA) depth profiles computed across the entire recording session for each animal (MUA was averaged on each channel for every second). Channels exhibiting the highest MUA amplitudes (yellow-red colors) were located in layer 5 (L5). For Rat 4, several channels were nonfunctional, and the signal-to-noise ratio was lower than in the other recordings; therefore, data from only three channels are shown (one from layer 2/3 and two from layer 5). Approximate layer borders are indicated next to the MUA depth profiles. L1-L6: layers 1-6.
